# Silicone Breast Implant Rupture From Pectoralis Muscle Contraction Causing IgA Nephropathy: A Case Report

**DOI:** 10.3389/fonc.2022.771409

**Published:** 2022-06-20

**Authors:** Mahno Noor Ezmas, Abdullah Norlia, Aziz Suraya, Wan Md Hafiz Wan Md Adnan, Lai Meng Looi

**Affiliations:** ^1^ Department of Surgery, Kulliyyah of Medicine, International Islamic University Malaysia, Kuantan, Malaysia; ^2^ Department of Surgery, Universiti Kebangsaan Malaysia Medical Centre, Kuala Lumpur, Malaysia; ^3^ Department of Radiology, Universiti Kebangsaan Malaysia Medical Centre, Kuala Lumpur, Malaysia; ^4^ Department of Internal Medicine, Universiti Malaya Medical Centre, Kuala Lumpur, Malaysia; ^5^ Department of Pathology, Universiti Malaya Medical Centre, Kuala Lumpur, Malaysia

**Keywords:** breast, carcinoma, pectoralis, contraction, silicone, implant, rupture, IgA nephropathy

## Abstract

A 34-year-old woman who was diagnosed with a left breast carcinoma underwent breast conserving surgery and axillary dissection. This was followed with adjuvant breast irradiation and endocrine therapy. She had a local recurrence in the breast 7 years later. She underwent a left nipple sparing mastectomy and submuscular implant reconstruction. The silicone implant ruptured during an episode of strong pectoralis muscle contraction, 5 years postimplantation. MRI confirmed the rupture to be intracapsular and extracapsular. She declined implant replacement. She presented with painless hematuria 2.5 years after the rupture. A renal biopsy confirmed IgA nephropathy.

## Introduction

This case reports pectoralis muscle contraction causing a submuscular breast implant rupture followed by IgA nephropathy in a woman with breast cancer. Silicone breast implant rupture is characterized by the breach of the implant shell (1, 2). Due to the large molecular weight of silicone, it is rarely found outside the shell, unless it has been breached (1, 2). There are two types of silicone implant rupture. The intracapsular is more common and is asymptomatic (1, 2). Extracapsular implant rupture is uncommon and may cause the patient to complain of breast pain or a mass in the breast (1, 2). It is probable that the incidence of intracapsular implant rupture is underreported, as it is silent. Most ruptures occur 10 years after implant placement (1, 2). The likelihood of rupture increases with implant age. The gold standard for the diagnosis is breast MRI (1, 2). The treatment option is a personalized decision, either conservative or surgical implant removal followed by replacement (1, 2). The diagnosis of IgA nephropathy (3, 4) is made through the analysis of a renal biopsy specimen. This is when IgA is the predominant immunoglobulin seen on the renal mesangium in immunofluorescence studies (3, 4). The possible autoimmune activation as a consequence of the continuous systemic contamination from a ruptured silicone implant is highlighted as a learning point in regard to conservative management of ruptured implants. On literature review, IgA nephropathy resulting from an *in situ* ruptured breast implant has not been clearly documented.

## Case Report

A 34-year-old woman, a Para 3, was diagnosed with a left breast carcinoma. She had undergone a breast conserving surgery (BCS) and axillary dissection (AD). It was followed by adjuvant breast irradiation and endocrine therapy (tamoxifen). At 41 years old, she had a 6-mm local recurrence in the breast, 7 years from the initial diagnosis. The tumor was estrogen and progestogen receptor positive. She underwent a left nipple sparing mastectomy (NSM) and immediate submuscular implant reconstruction. She had declined an autologous breast reconstruction. This is because she did not want a secondary defect with its associated donor site morbidities. She was given subcutaneous depot Goserelin, a luteinizing hormone-releasing hormone (LHRH) analogue, and anastrozole, an aromatase inhibitor (AI), for 2 years followed by tamoxifen for 1 year. She discontinued endocrine treatment, as she developed menorrhagia on tamoxifen. She did not wish to continue on an LHRH suppression/AI and declined oophorectomy.

The breast implant used was a textured, round double-lumen silicone with saline expander. It was a Siltex Round Becker 25 Cohesive I breast implant that was 25% cohesive silicone gel in the outer lumen with 75% liquid (saline or water) in the inner lumen. The implant contained 50 cc silicone gel in the outer lumen with a deflated (empty) inner lumen. It was placed submuscular to the pectoralis major and serratus anterior. She had several clinic visits for infusion of sterile water; 25–50 cc was injected at every session into the subcutaneous port for gradual expansion of the inner lumen. A total of 125 cc of water was the final amount injected. The manufacturer’s recommended volume was 125–200 cc. There was asymmetry, with the reconstructed breast smaller than the contralateral side, which was also more ptotic. She had declined implant cover with the latissimus dorsi flap. She also declined surgery on the contralateral side. She was otherwise fairly symmetrical in a bra. The implant port just below the left inframammary region was removed uneventfully later. Clinically, there was no obvious capsular contracture seen on her follow-up assessments. Five years after her left breast reconstruction, she complained of left chest discomfort, left shoulder pain with limited range of movement, and a painful swelling in the left axilla for a duration of 2 weeks. Upon recall, she said that the problem occurred a few days after partially lifting a large television set by herself, using both arms. It was associated with fever, erythema of the left upper chest wall, left proximal upper arm swelling, and tender left axillary nodes. She also complained of painful left shoulder abduction and was able to abduct to a maximum of 60 degrees only.

**Figure 1:**
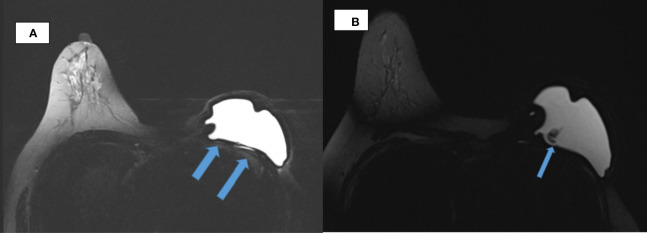
Breast MRI. **(A)** Axial TIRM image of MR breasts demonstrates irregularity of the silicone implant shell with inward invagination in the left breast. Linear hyperintensity that is of fluid intensity noted posterior to the implant till its lateral border raises the suspicion of an extracapsular rupture (arrowed). **(B)** Wavy lines (arrowed) evident within the silicone implant giving the linguine sign, which suggest an intracapsular rupture.

Ultrasound of the left arm and axilla showed focal interstitial edema of the medial part of the left upper arm with several reactive axillary lymph nodes. MRI of the breast demonstrated an irregular shell with a posterior hyperintense subcapsular line. There was invagination of the silicone membrane with a droplet of silicone within, giving the appearance of the noose sign. There was also a linguine sign seen. No enlarged axillary nodes were detected (**Figure 1**). A PET-CT was deemed unnecessary to assess the lymph nodes, as they were already adequately assessed *via* ultrasound. Ultrasound is the best imaging modality to assess axillary lymph nodes.

Her symptoms resolved with antibiotics and non-steroidal anti-inflammatory drugs (NSAIDs). She declined implant removal or replacement, autologous reconstruction, and contralateral symmetrization surgery but remained on regular follow-up. As no biopsy or surgical procedure was performed after the implant rupture, histological examination of the periprosthetic capsule could not be performed.

She was able to do most regular movements using her left arm. However, often, she would experience sudden severe pain causing “locking” of the arm if she did posterior extension beyond 135 degrees or if she fully stretched her left arm upwards above her head. Then, she would have to rotate her left arm back and forth slowly in short circles, to enable it to become “unlocked.”

About 2.5 years after the implant rupture, she developed an episode of upper respiratory tract infection. A week later, she noticed gross painless hematuria. She consulted a nephrologist. Her blood pressure was elevated, and blood results showed derangement of renal function, with serum creatinine of 110 μmol/L (normal, 44–71 μmol/L), and she was passing 4 g of proteinuria per day (normal, <0.15 g/24 h). The IgA was slightly elevated at 407 mg/dl (normal, 70–400 mg/dl), but antinuclear antibody (ANA) and antineutrophil cytoplasmic antibody (ANCA) were negative. The complement level and antistreptolysin antibody were normal as well.

**Figure 2:**
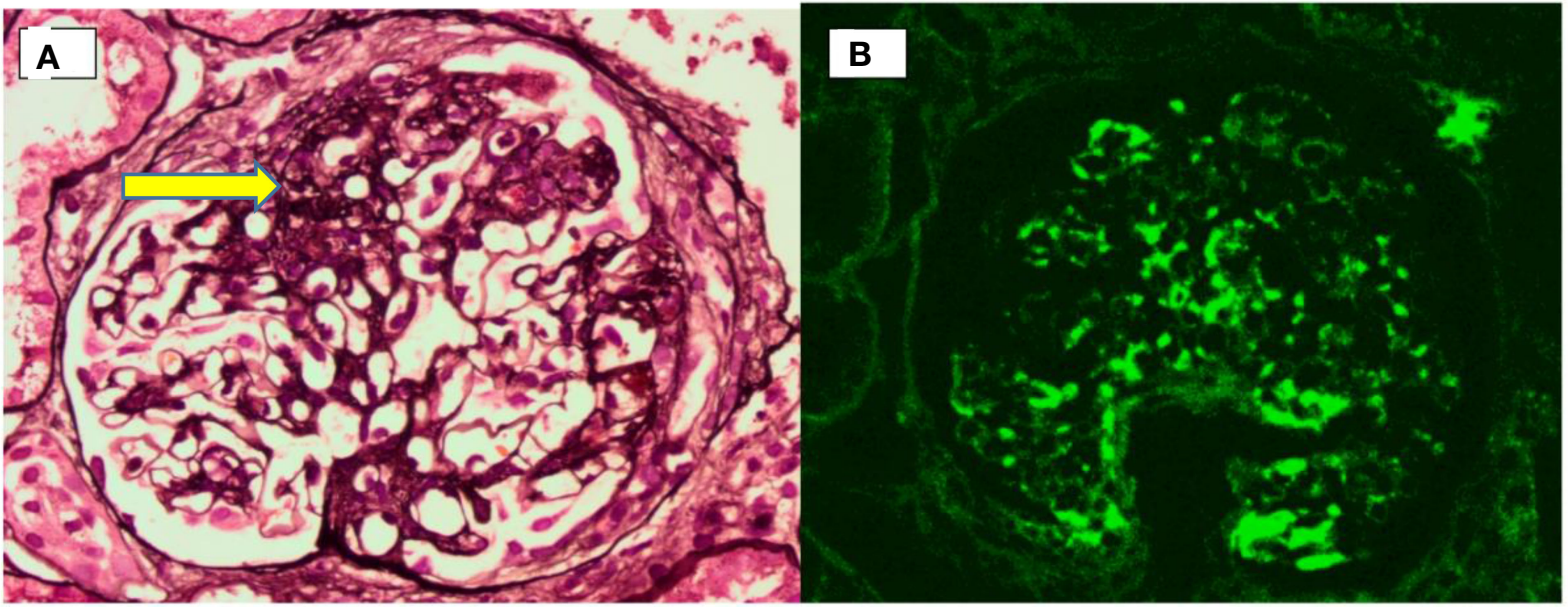
Renal biopsy. **(A)** Photomicrograph showing an area of segmental sclerosis (arrow) within a glomerulus. **(B)** Immunofluorescence microscopy showing predominant mesangial IgA deposition.

**Figure 3:**
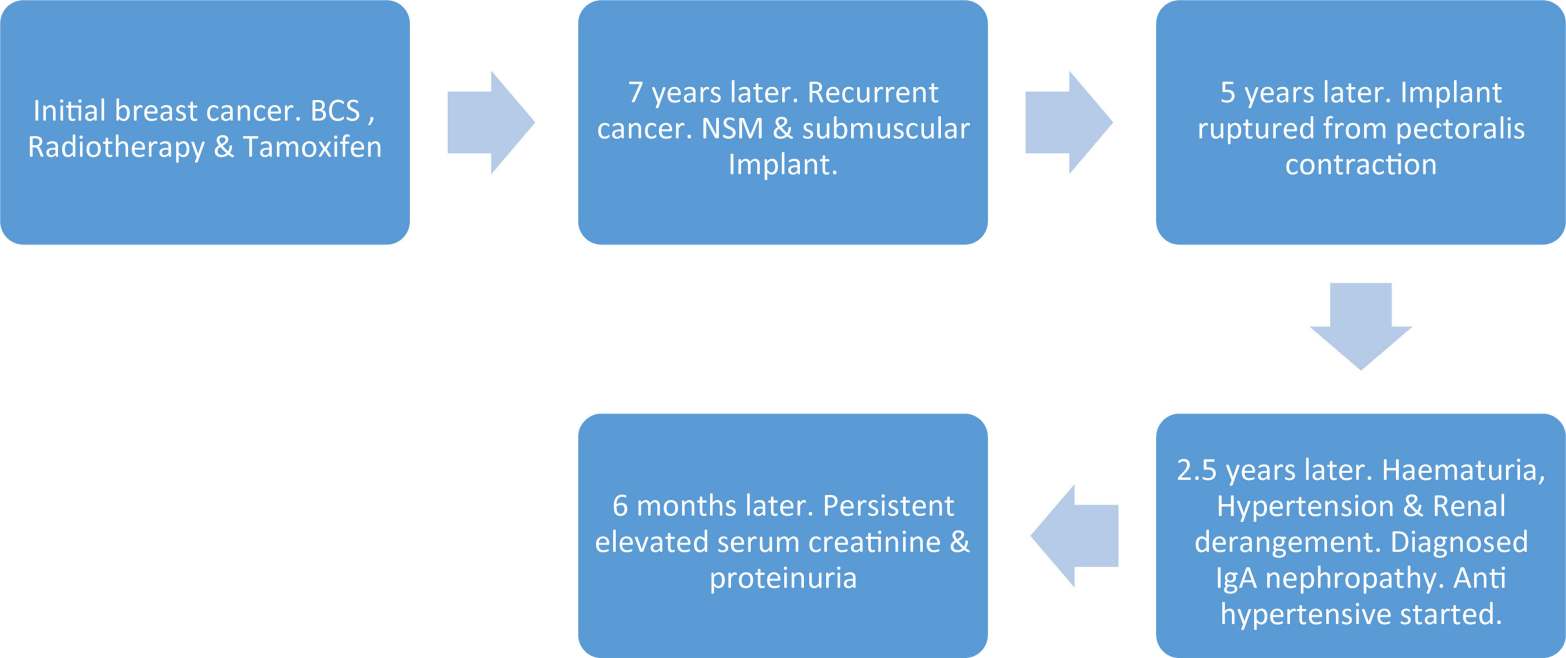
Timeline of events. BCS, breast-conserving surgery; NSM, nipple-sparing mastectomy.

A renal biopsy captured 23 glomeruli, of which 1 was globally sclerosed and 4 showed segmental sclerosis (**Figure 2A**). The remaining glomeruli appeared morphologically within normal limits. Immunofluorescence examination revealed heavy deposits of IgA in the mesangium (**Figure 2B**) with smaller amounts of C3. A diagnosis of IgA nephropathy with a focal segmental glomerulosclerosis pattern was made. She was started on an angiotensin receptor blocker. Her latest laboratory results 6 months after the renal biopsy showed a lowered serum creatinine level of 89 µmol/L (normal, 44–71 μmol/L) and 24-h urinary protein of 0.8 g (normal, <0.15 g/24 h). Refer **Figure 3** for the timeline of events in this patient.

## Discussion

Implant reconstruction is preferred over autologous tissue reconstruction for small and moderate breast sizes because of its relative ease and short operative time (1). Although more costly, permanent inflatable implants are more advantageous than temporary implants or fixed size implants. This is due to better cosmetic outcome, as the volume is adjustable to achieve the desired target (1). Furthermore, there is minimal discomfort felt by the patient, as the expansion is gradual in contrast to a fixed size implant. However, the patient would need to make several clinic visits postoperatively, and the port would require removal as a day case procedure.

Being a foreign material, the breast implant tends to be gradually walled off by the body with a surrounding fibrous capsule or shell, which is known as capsular formation (1). The amount of capsule formed may vary from mild, moderate, or severe. If the amount of fibrous tissue formed is excessive, it is known as capsular contracture (1). This is often related to external beam radiotherapy postimplant placement, and the hardened tissue may cause pain and deformity (1, 2).

An implant rupture is clinically defined as the breach of any size in the implant shell (1). Silicone implant rupture is divided into intracapsular and extracapsular (2). For intracapsular tears, free silicone remains contained within the surrounding fibrous capsule (1). The extracapsular type is the rupture of both implant shell and outer fibrous capsule. This causes silicone leakage into the surrounding tissues and even embolization to distant sites (1). For this patient, she had both an intracapsular and extracapsular implant rupture.

The incidence of implant rupture ranges widely from 0.3% to 77% in general and remains a controversial issue (1). It is difficult to compare the results of many cross-sectional rupture prevalence studies because of the rapid growth of many generations of implants by different manufactures, different follow-up periods, and no standard diagnostic imaging due to limited resources (1). The recent generation of implants are expected to have less capsular contracture, with rupture rates between 12% and 15% (2). Based on a 6-year analysis, the rate of rupture of expandable breast implants is 1.5% in an MRI cohort study (5). There are no current data comparing the rates of rupture of the latest generation of fixed size silicone versus expandible implants.

The mechanism of rupture can be multifactorial. Spontaneous rupture is more common than that caused by direct blunt trauma to the implant (2). Based on the analysis of 30 implant ruptures, only one case involving a double lumen implant was reported. This implied that such implants had a low rupture rate (5). However, this could also be because more fixed size than expandible implants are used due to their lower cost, wider availability, and less postoperative care.

Early implant rupture during the first 2 years is usually due to inadvertent damage to the implant, such as physical trauma to elastomeres at the time of implantation (1, 6). The development of capsular contracture or fold-flaw failure increases with implant age, usually after 10 years (1, 2). Thus, the average implant age to undergo rupture is approximately 8 years (1, 2, 6). For mechanical injuries, there have been reports of the causative factor being compression related to mammogram or blunt trauma (1, 2). She had left breast radiation therapy in 2006; this may have led to the pectoral muscles to be more hardened and fibrotic.

However, there is no long-term conclusive data on implant rupture rate and the possible influence or correlation to radiotherapy. The mechanism of trauma resulting from strong contraction of the pectoralis muscle causing implant rupture has not been previously reported.

The clinical diagnosis of an implant rupture is made by the respective surgeon in approximately 30% of cases, especially through a good history and physical examination (1). Most symptoms reported are pain, a palpable lump, or skin changes. Most signs reported are contour deformity (44%), implant displacement (20%), and a palpable mass (17%) (1). For our patient, the suspicion of implant rupture was a clinical diagnosis by the surgeon, due to the lateral deviation of her nipple areolar complex. This was confirmed by magnetic resonance imaging (MRI) of the breast. MRI is considered as the gold standard for diagnostic imaging of implant rupture due to its high sensitivity (80%–90%) and specificity (90%–97%) (1, 2).

Explantation is the gold standard treatment for a ruptured silicone implant. However, it is still a personalized decision (1, 2). The discussion between the patient and surgeon regarding the possible complications should be addressed.

Two and a half years after the implant rupture, she had a short episode of fever and sore throat followed by painless hematuria. She was found to have hypertension, renal impairment and anemia. Renal biopsy confirmed the diagnosis of IgA nephropathy.

IgA nephropathy is the most common primary glomerulonephritis worldwide (3, 4). It is characterized by dominant IgA deposits in the glomeruli, accompanied by histological changes such as mesangial or endocapillary hypercellularity, segmental glomerulosclerosis, or crescent formation (3, 4). The diagnosis of IgA nephropathy has been documented in patients with a variety of co-morbidities such as liver disease caused by infection with hepatitis B, hepatitis C, or human immunodeficiency virus (HIV) and infections such as malaria and Lyme disease (4). It has also been reported in autoimmune disorders such as systemic lupus erythematosus (SLE), rheumatoid arthritis, Sjogren’s syndrome, psoriasis, and malignancy such as lymphoma (7). IgA nephropathy can be present in 4%–16% of the population (7) from unknown causes, deemed idiopathic or primary in origin. Due to this, the association between IgA nephropathy and coexisting disease may simply be coincidental. To date, there are no specific histological features differentiating primary from secondary IgA nephropathy. In our case, we propose that the patient’s occurrence of IgA nephropathy is related to the continuous systemic silicone contamination from her ruptured breast implant.

This is more so, as there has been continuous silicone leakage systemically for the past 20 months in this patient. Since the diagnosis of IgA nephropathy 10 months ago, her blood pressure has been controlled with an antihypertensive. However, her proteinuria and hematuria have not stopped. This is likely because the source of the systemic inflammation involving the renal system, which is the ruptured breast implant, has remained *in situ*.

Previously, silicone gel breast implants have been associated with a myriad of autoimmune and connective tissue disorders by anecdotal reports and small observational studies. These studies were in women who had implants for cosmetic reasons and cancers (8, 9) To date, no increased risk of connective tissue disease has been detected in ruptured implants (1, 2, 6).

Although there has been no report linking silicone rupture and IgA nephropathy, the silicone degradation product produced by a ruptured implant may activate both the innate and the adaptive immune system cells, leading to a chronic inflammatory process. In fact, dendritic cells, macrophages, fibroblast, and T cells have all been found at the capsule/silicone implant contact zone (10). A study involving 520 women with silicone breast implants showed significantly higher levels of IgM, IgG, IgA, and IgE antisilicone antibodies compared to controls (11). The circulating IgG–IgA complexes may deposit and trigger local inflammation in the glomeruli and predispose to the development of IgA nephropathy. Previously, it has been reported that ruptured silicone may have renal consequences, such as secondary renal amyloidosis (12) and scleroderma renal crisis (13), and may even contribute to allograft kidney rejection (14).

Siliconosis has also been proposed as a possible cause of chronic kidney disease (15).

Since silicone is hydrophobic, most ruptured materials remain adherent to the implant surface. It is unlikely to be transported by any mechanism other than macrophage migration or local diffusion to the nearby nodes leading to reactive lymphadenopathy (16). As a foreign body, as long as it is not removed, it could induce recurrent and a chronic inflammatory reaction.

## Conclusion

This case of a ruptured submuscular implant from overexertion of the pectoralis muscle has not been reported before. Postoperatively, such patients may need to be advised against strenuous physical activities involving the upper limb as a prophylactic measure to prevent implant ruptures. Secondary IgA nephropathy due to an autoimmune reaction triggered by an implant rupture is a possible correlation. This patient may benefit from removal and replacement of the implant. She should also consider an autologous breast reconstruction instead, which is free of implants. By doing so, her renal function may return to normality.

## Data Availability Statement

The raw data supporting the conclusions of this article will be made available by the authors, without undue reservation.

## Ethics Statement

Written informed consent was obtained from the individual(s) for the publication of any potentially identifiable images or data included in this article.

## Author Contributions

ME: drafted the document. AN: conceptualized this case report, revised and edited the document, obtained the patient’s consent, provided photos of the patient, and was the primary clinician. AS: performed and reported the radiological images. WW: the nephrologist treating the patient and provided information in regard to the pathophysiology of the patient’s IgA nephropathy. LL: prepared the renal biopsy images and provided pathophysiology information of the patient’s IgA nephropathy.

## Conflict of Interest

The authors declare that the research was conducted in the absence of any commercial or financial relationships that could be construed as a potential conflict of interest.

## Publisher’s Note

All claims expressed in this article are solely those of the authors and do not necessarily represent those of their affiliated organizations, or those of the publisher, the editors and the reviewers. Any product that may be evaluated in this article, or claim that may be made by its manufacturer, is not guaranteed or endorsed by the publisher.

## References

[1] Urban C. “Implant Rupture”. In: Urban C, editor. Oncoplastic and Reconstructive Breast Surgery. Cham: Springer International Publishing AG (2019). p. 610–9. Available at: 10.1007/978-3-319-62927-8_49.

[2] Hillard C Fowler JD Barta R. Cunningham B. Silicone Breast Implant Rupture: A Review. Gland Surg (2017) 6:163–8. doi: 10.21037/gs.2016.09.12 PMC5409893 28497020

[3] Peh SC Looi LM Wang F Chua CT Tan HW Lam KL. The Histopathological Pattern of Primary IgA Nephropathy in a Malaysian Patient Population. Malaysian J Pathol (1990) 12(1):21–6. 2090886

[4] Looi LM. The Pattern of Renal Disease in Malaysia. Malaysian J Pathol (1994) 16(1):19–21. 16329571

[5] Hammond DC Migliori MM Caplin DA Garcia ME Philips CA. Mentor Contour Profile Gel Implants: Clinical Outcomes at 6 Years. Plast Reconstr Surg (2012) 129:1381–1391. doi: 10.1097/PRS.0b013e31824ecbf0 22327894

[6] Handel N Garcia ME Wixtrom R. Breast Implant Rupture: Cause, Incidence, Clinical Impact and Management. Plast Reconstr Surg (2013) 132:1128–37. doi: 10.1097/PRS.0b013e3182a4c243 24165596

[7] Saha MK Julian BA Novak J Rizk DV. Secondary IgA Nephropathy. Kidney Int (2018) 94:674–81. doi: 10.1016/j.kint.2018.02.030 PMC6981247 29804660

[8] Berner I Gaubitz M Jackisch C Pfleiderer B. Comparative Examination of Complaints of Patients With Breast Cancer With and Without Silicone Implants. Eur J Obstet Gynecol Reprod Biol (2002) 102:61. doi: 10.1016/S0301-2115(01)00561-9 12039092

[9] Gaubitz M Jackisch C Domschke W Heindel W Pfleiderer B. Silicone Breast Implants: Correlation Betweenimplant Ruptures, Magnetic Resonance Spectroscopically Estimated Silicone Presence in the Liver, Antibody Status and Clinical Symptoms. Rheumatology (2002) 41:129. doi: 10.1093/rheumatology/41.2.129 11886959

[10] Wolfram D Rainer C Niederegger H Piza H Wick G. Cellular and Molecular Composition of Fibrous Capsules Formed Around Silicone Breast Implants With Special Focus on Local Immune Reactions. J Autoimmun (2004) 23:81–91. doi: 10.1016/j.jaut.2004.03.005 15236756

[11] Vojdani A Brautbar N Campbell AW. Antibody to Silicone and Native Macromolecules in Women With Silicone Breast Implant. Immunopharmacol Immunotoxicol (1994) 16:497–523. doi: 10.3109/08923979409019737 7533175

[12] Emekli U Tumerdem B Demiryont M. Rupture of a Silicone Gel Mammary Prosthesis and Amyloidosis: A Case Report. Aesthetic Plast Surg (2002) 26:383–7. doi: 10.1007/s00266-002-2022-x 12432480

[13] Al Aranji G White D Solanki K. Scleroderma Renal Crisis Following Silicone Breast Implant Rupture: A Case Report and Review of Literature. Clin Exp Rheumatol (2014) 32:262–66. doi: 10.1007/s12026-016-8871-1 24480575

[14] Basic-Jukic N Ratkovic M Radunovic D Kastelan Z. Association of Silicone Breast Implants and Acute Allograft Rejection. Med Hypotheses (2019) 123:81–2. doi: 10.1016/j.mehy.2018.12.012 30696600

[15] Barilaro G Testa CS Caciani A Donato G Dimko M Mariotti A. ASIA Syndrome, Calcinosis Cutis and Chronic Kidney Disease Following Silicone Injection. A Case-Based Review. Immunol Res (2016) 64:1142–9. doi: 10.1007/s12026-016-8871-1 27665458

[16] Major MR Wong VW Nelson ER Longaker MT Gurtner GC. The Foreign Body Response: At the Interface of Surgery and Bioengineering. Plast Reconstr Surg (2015) 135:1489–98. doi: 10.1097/PRS.0000000000001193 25919260

